# Vaccination against COVID-19: the view of Brazilian federal judges

**DOI:** 10.1590/0102-311XEN086823

**Published:** 2024-04-29

**Authors:** Wilson Medeiros Pereira, Fabrício Emanuel Soares de Oliveira, Maressa Lopes Coelho, Daniella Reis Barbosa Martelli, Hercílio Martelli

**Affiliations:** 1 Universidade Estadual de Montes Claros, Montes Claros, Brasil.; 2 Tribunal Regional Federal da 6ª Região, Belo Horizonte, Brasil.; 3 Centro Pró-Sorriso, Universidade de Alfenas, Alfenas, Brasil.

**Keywords:** COVID-19, Vaccination, Obligatory Vaccination, Judiciary, COVID-19, Vacinação, Vacinação Obrigatória, Poder Judiciário, COVID-19, Vacunación, Vacunación Obligatoria, Poder Judicial

## Abstract

The aim was to analyze the perception of Brazilian federal judges on the implications of COVID-19 vaccination. A study was carried out with Brazilian federal judges, who received a survey designed with multiple-choice questions on COVID-19 vaccination, covering topics such as its mandatory aspect, the application of coercive measures, hesitation to vaccinate, priority groups, the duties of Brazilian Health Regulatory Agency (Anvisa, acronym in Portuguese), the role of the Judiciary branch, and immunity passports. A total of 254 out of 1,300 federal judges from all states responded to the survey. Most respondents have a Bachelor’s degree or a specialization (59.1%) and have been judges for more than 10 years (63.8%). A great majority of the judges (87.7%) agree with vaccine mandates for adults and for children and adolescents (66.1%). Over 75% of judges believe that all levels of government can impose sanctions on those who refuse to get vaccinated. The judges trust vaccination 93% of the time, 56.1% reject anti-vaccination movements, and 75.2% believe that Anvisa duties should be respected. The Judiciary branch actions concerning the COVID-19 pandemic are approved by 62.6% of judges, and 88.2% support immunity passports. There is a direct connection among mandatory vaccination, trust in the vaccine, and the adoption of immunity passports. Most federal judges agree with vaccine mandates for children and adults, support the application of sanctions for vaccination refusal, disapprove of anti-vaccination movements, agree with Anvisa’s duties, and support judicial intervention in relation to the COVID-19 pandemic.

## Resumo

O objetivo foi analisar a visão de juízes federais brasileiros sobre as implicações da vacinação contra a COVID-19. Foi realizado um estudo com juízes federais brasileiros, que receberam uma pesquisa elaborada com questões de múltipla escolha sobre a vacinação contra a COVID-19, abordando temas como sua obrigatoriedade, aplicação de medidas coercitivas, hesitação vacinal, grupos prioritários, ações da Agência Nacional de Vigilância Sanitária (Anvisa), o papel do Poder Judiciário e os passaportes de imunidade. Responderam à pesquisa 254 dos 1.300 juízes federais atuantes em todos os estados. A maioria dos entrevistados possui bacharelado ou especialização (59,1%) e atua como juiz há mais de 10 anos (63,8%). Grande parte dos juízes (87,7%) concorda com a obrigatoriedade da vacina para adultos e crianças e adolescentes (66,1%). Mais de 75% dos juízes acreditam que todos os níveis de governo podem impor sanções para aqueles que se recusam a ser vacinados. 93% dos juízes confiam na vacinação, 56,1% rejeitam movimentos antivacinação e 75,2% acreditam que as ações da Anvisa devem ser respeitadas. As ações do Judiciário referentes à pandemia da COVID-19 são aprovadas por 62,6% dos juízes e 88,2% apoiam passaportes de imunidade. Existe uma ligação direta entre a vacinação obrigatória, a confiança na vacina e a adoção de passaportes de imunidade. A maioria dos juízes federais concorda com a obrigatoriedade da vacina para crianças e adultos, apoia a aplicação de sanções no caso de recusa de vacinação, desaprova movimentos antivacinação, concorda com as ações da Anvisa e apoia a intervenção do Judiciário em relação à pandemia da COVID-19.

## Resumen

El objetivo fue analizar la visión de los jueces federales brasileños sobre las implicaciones de la vacunación contra la COVID-19. Se realizó un estudio con jueces federales brasileños, quienes recibieron una encuesta elaborada con preguntas de opción múltiple sobre la vacunación contra la COVID-19, abordando temas como su obligatoriedad, aplicación de medidas coercitivas, reticencia a vacunarse, grupos prioritarios, acciones de Agência Nacional de Vigilancia Sanitaria (Anvisa), el papel del Poder Judicial y los pasaportes de inmunidad. Respondieron a la encuesta 254 de los 1.300 jueces federales que actúan en todos los estados. La mayoría de los encuestados tiene título de licenciatura o especialización (59,1%) y actúa como juez desde hace más de 10 años (63,8%). Gran parte de los jueces (87,7%) está de acuerdo con la obligatoriedad de la vacuna para adultos y niños y adolescentes (66,1%). Más del 75% de los jueces cree que todos los niveles de gobierno pueden imponer sanciones a quienes se nieguen a vacunarse. El 93% de los jueces confía en la vacunación, el 56,1% rechaza los movimientos antivacunas y el 75,2% cree que las acciones de Anvisa deben ser respetadas. Las acciones del Poder Judicial con relación a la pandemia de COVID-19 son aprobadas por el 62,6% de los jueces, y el 88,2% apoya los pasaportes de inmunidad. Existe un vínculo directo entre la vacunación obligatoria, la confianza en la vacuna y la adopción de pasaportes de inmunidad. La mayoría de los jueces federales está de acuerdo con la vacunación obligatoria para niños y adultos, apoya la aplicación de sanciones en caso de rechazo de la vacunación, desaprueba los movimientos antivacunas, está de acuerdo con las acciones de Anvisa y apoya la intervención del Poder Judicial con relación a la pandemia de COVID-19.

## Introduction

The outbreak of a pandemic can affect the entire society, both in the public and private sectors. It holds huge consequences, and measures need to be taken to deal with the crisis and come up with strategies to overcome it ^1^. The possibility of access to a vaccine can bring peace of mind to the population and allows them to see life returning to normal. The first vaccines approved by the Brazilian Health Regulatory Agency (Anvisa, acronym in Portuguese) in Brazil were CoronaVac (Butantan Institute), Oxford/Covishield (Fiocruz and Astrazeneca), Janssen Vaccine (Janssen-Cilag), and Comirnaty (Pfizer/Wyeth).

When the process of immunization began in the country, there was a limited supply of doses available to the population, which led to a massive demand for vaccines. That situation led to all sorts of problems, both in big cities and small towns, such as misappropriation of vaccines ^2^, people who didn’t belong to the priority groups being vaccinated out of turn ^3^, a declaration of public calamity in some states ^4^, criticism of the actions of the Brazilian Ministry of Health ^5^, and exposure of the identity of vaccinated people ^3^. Over time, vaccination rates increased. By March 2023, over 70% of the world’s population had received at least one dose of the COVID-19 vaccine ^6^.

Themes such as the application of coercive measures, the freedom to oppose vaccination campaigns, the privacy of vaccinated people, the identification of priority groups, and the commercialization of vaccines by the private sector have repeatedly been subject for debate in Brazil ^7^. At the end of 2020, the Brazilian Supreme Federal Court (STF, acronym in Portuguese) ruled that vaccination should not be forced, which gives citizens the right to refuse it. However, the Court understands that certain sanctions could be applied, such as restrictions on some activities or bans from schools or other public spaces ^8^. The requirement of immunity passports is another relevant theme. The passport must record the vaccination against COVID-19 and is like the International Certificate of Vaccination or Prophylaxis (ICVP) ^9^ of the World Health Organization (WHO). This certificate acts as proof that the bearer has been vaccinated against cholera, plague, typhoid fever, among other diseases.

Each of the aforementioned issues has been brought before the Judiciary branch for consideration. Most of those cases involve the Brazilian Ministry of Health or Anvisa, which are federal entities, so the whole process often falls under the responsibility of the Federal Justice. During the COVID-19 crisis, Brazilian courts have tried cases concerning public health policies, in view of questionable actions taken by the Executive branch ^10^. Thus, it is rather relevant to know the perceptions of federal judges on matters related to the pandemic, since judicial decisions can give a different direction to public policies, such as those related to health. To this end, this paper aims to study the thinking of Brazilian federal judges on the ramifications of the COVID-19 vaccine.

## Materials and methods

Cross-sectional study and census method were used to approach Brazilian federal judges in the five Federal Regional Courts (TRFs, acronym in Portuguese) (Federal District: https://www.trf1.jus.br/trf1/home/; Rio de Janeiro/Espírito Santo: https://www10.trf2.jus.br/portal/; São Paulo/Mato Grosso do Sul: https://www.trf3.jus.br/; Rio Grande do Sul/Santa Catarina/Paraná: https://www.trf4.jus.br/trf4/controlador.php?acao=principal&; and Alagoas/Ceará/Paraíba/Pernambuco/Rio Grande do Norte/Sergipe: https://www.trf5.jus.br/index.php).The survey for this study was designed with 19 multiple-choice questions about the implications of the COVID-19 pandemic and was based on the following variables: (1) demographic: place of work (in which state), level of education without a specialization degree or with a specialization or graduate degree; work experience (up to 10 years, from 10 to 20 years, or more than 20 years); (2) vaccination: its mandatory aspect (for children and adults), the legal role - of the Federal Government, of any federal entity, or both - in applying sanctions for those who refuse the vaccine, anti-vaccination movements, trust in vaccination, control of vaccinated people, designation of doses for specific groups, sanctions for non-compliance with vaccination orders, vaccine hesitancy, purchase of vaccine by the private sector; (3) the roles of the Judiciary branch and Anvisa, and the requirement for an immunity passport.

This research involves federal judges’ stances regarding the vaccination against COVID-19. All vaccines used in the country must be pre-approved by Anvisa, which is an autonomous government agency created by *Law n. 9,782* of January 26, 1999 ^11^. The *Brazilian Federal Constitution* ^12^ states, according to its article 109, that cases involving federal-level entities must be prosecuted by the Federal Justice. During the pandemic, the STF authorized state and municipal governments to establish their own restriction protocols, which gave federal judges the power to try related cases.

The SurveyMonkey platform (https://www.surveymonkey.com/) was used to send the survey questions individually to the official email addresses of each federal judge, respecting their privacy. The survey was sent out to 1,300 federal judges, who were selected according to their field of expertise, that is, those acting in general jurisdiction or in civil or small claims court. Judges acting exclusively in the Environmental, Criminal, Criminal Organizations, International, and Social Security areas were not considered for the survey.

Once the responses to the survey had been received, we were able to establish a database and carry out the statistical analysis using SPSS software version 18.0 (https://www.ibm.com/). The variables were analyzed in their absolute and relative frequencies with chi-squared test, considering that they were categorical variables. This research was submitted to and approved by the Research Ethics Committee of the State University of Montes Claros (# 5,032,572). Each federal judge had to sign a free and informed consent form.

## Findings

Of the 1,300 survey forms sent out to as many federal judges as possible, a total of 254 (19.53%) were answered by judges working in all states of the country, most of them in the states of Minas Gerais (n = 56; 22%), Bahia (n = 28; 11%), and São Paulo (n = 21; 8.3%). The majority of respondents (n = 150; 59.1%) do not have or have concluded a specialization degree. Regarding their career experience, 63.8% (n = 162) have been judges for more than 10 years (Table 1).

**Table 1:** Sociodemographic profile of Brazilian federal judges (n = 254).

Characteristics	n	% (95%CI)
Region		
North	29	11.4 (7.9-15.7)
Northeast	54	21.3 (16.5-26.6)
Central-West	33	13.0 (9.2-17.5)
Southeast	87	34.3 (28.6-40.2)
South	51	20.1 (15.5-25.3)
Level of education		
No specialization/Some specialization degree	150	59.1 (52.9-65.0)
Master’s degree/Doctoral degree/Post-doctoral degree	104	40.9 (35.0-47.1)
Career experience (years)		
< 10	92	36.2 (30.5-42.2)
≥ 10	162	63.8 (57.8-69.5)

95%CI: 95% confidence interval.

Vaccine mandate, as established by the STF (compulsory, but not forced), was approved by 87.7% (n = 221) of respondents. On the other hand, the immunization of children and adolescents, with or without parental consent, had a lower approval rate, 66.1% (n = 167). Regarding the prerogative to establish coercive measures for non-compliance with vaccination, 75.2% (n = 191) believe that the Federal Government should not be the only party to have such power. State and local governments should also have the power to establish punishment procedures.

Trust in vaccination is high among judges, with a rate of 93.3% (n = 237). Regarding anti-vaccination movements, most judges (n = 142; 56.1%) think that anti-vaccination campaigns should be forbidden, since they go against public interest. Concerning the obstacles to vaccination in the country, 86.3% (n = 217) believe that the public authorities’ rhetoric questioning the effectiveness of the vaccine and the widespread dissemination of fake news have had a significant impact. When it comes to Anvisa’s duties in analyzing vaccines, 75.2% of judges (n = 188) think that the agency’s legal and administrative procedures should be followed properly. In relation to the vaccination against COVID-19, 62.6% (n = 159) of respondents agree with the actions of the Judiciary branch. Finally, 88.2% (n = 223) of federal judges approve the adoption of immunity passports in Brazil and believe that each state in the nation can establish their own requirements to regulate it (Table 2).

**Table 2:** Vaccine mandates for children and adults, anti-vaccination campaigns, application of sanctions, the role of Brazilian Health Regulatory Agency (Anvisa), actions of the Judiciary branch, and use of immunity passport (n = 254).

Characteristics	n	% (95%CI)
Approval of mandatory vaccination against COVID-19		
Yes, but without physical coercion	221	87.7 (83.1-91.2)
No, individual freedom must be respected	31	12.3 (8.6-16.7)
Childhood vaccination		
Vaccination must be mandatory, even without parental consent	167	66.0 (60.0-71.7)
Parents’ and guardians’ decision must be respected	86	34.0 (28.3-40.0)
Sanctions or measures for vaccine non-compliance		
Only if established by the federal government	63	24.8 (19.8-30.4)
States, the Federal District, and municipalities may also establish them	191	75.2 (69.6-80.2)
Anti-vaccination campaigns		
Must be forbidden	142	56.1 (50.0-62.2)
I support them, but with no endorsement of public authorities	82	32.4 (26.8-38.3)
I support them, due to the right to freedom of speech	29	11.5 (7.9-15.8)
Trust in vaccination against COVID-19		
Yes, as I trust any other vaccine	237	93.3 (89.8-96.0)
No, there is no scientific evidence of its effectiveness	6	2.4 (0.9-4.7)
No, for different reasons	11	4.3 (2.3-7.3)
Disclosure of the identity of vaccinated people for control		
Yes	139	54.7 (48.6-60.8)
No	115	45.3 (39.2-51.4)
Designation of doses for specific groups, even if not considered as priority		
Yes	35	13.8 (9.9-18.4)
No	219	86.2 (81.6-90.1)
Sanctions for those who refuse the vaccine		
Must be pecuniary	215	86.0 (81.3-89.9)
In case of multiple-dose vaccines, denial of next dose	10	4.0 (2.0-7.9)
No sanction must be applied, as it is an administrative violation	25	10.0 (6.7-14.1)
Person to be punished for vaccine non-compliance		
The citizen	31	12.3 (8.6-16.7)
The authority or agency that gave authorization	12	4.8 (2.6-7.9)
Both	209	82.9 (78.0-87.2)
Obstacles to the vaccination against COVID-19		
Public authorities’ speech questioning the effectiveness of the vaccine	113	44.5 (39.3-51.6)
People’s distrust in laboratories	21	8.4 (5.4-12.3)
Anti-vaccination campaigns	104	41.8 (35.7-48.0)
Distrust about the source of the disease	11	4.4 (2.3-7.4)
Purchase of vaccines by private parties		
I am against it	146	58.2 (52.0-64.2)
I am in favor of it	105	41.8 (35.8-48.0)
Anvisa’s duties to analyze vaccines		
The agency’s legal and administrative procedures should be followed	188	75.2 (69.6-80.3)
Approval by expiration of deadline can be justifiable in case of delays	45	18.0 (13.6-23.1)
There may be political intervention in the agency if there is a need to speed up the analysis of vaccine proposals	17	6.8 (4.1-10.4)
Actions of the Judiciary branch in the context of the COVID-19 vaccination		
The Judiciary branch can interfere in exceptional cases	149	58.7 (52.5-64.6)
The Judiciary branch must restrain itself and prioritize administrative decisions	95	37.4 (31.6-43.5)
The Judiciary branch can always interfere	10	3.9 (2.0-6.8)
Use of “immunity passport” in Brazil		
I agree, as long as implemented by federal law	94	37.2 (31.4-43.2)
I agree, and any level of the government can establish their own requirements	129	51.0 (44.8-57.1)
I disagree	30	11.9 (8.3-16.2)

95%CI: 95% confidence interval.

Tables 3 and 4 show the association between the variables. According to the results, there is a considerable relation between those in favor of the vaccine mandate and the vaccination of children and adolescents. The results also show that work experience and level of education affect the judges’ perceptions on vaccination and reveal that there is a connection between being in favor of vaccine mandates, trust in vaccination, and prohibition of purchasing by private parties.

**Table 3:** Comparison of variables regarding vaccination, level of education, and career experience (n = 254).

Characteristics	Level of education		Career experience (years)		
	No specialization degree/Some specialization degree	Master’s degree/Doctoral degree/Post-doctoral degree	p-value	< 10	≥ 10	p-value
	n (%)	n (%)		n (%)	n (%)	
Mandatory vaccination against COVID-19						
Yes, but without physical coercion	133 (88.7)	88 (86.3)		81 (90.0)	140 (86.4)	
No, individual freedom must be respected	17 (11.3)	14 (13.7)	0.674 *	9 (10.0)	22 (13.6)	0.058 *
Childhood vaccination						
Must be mandatory, even without parental consent	96 (64.0)	71 (68.9)		64 (70.3)	103 (63.6)	
Parents’ and guardians’ decision must be respected	54 (36.0)	32 (31.1)	0.416 **	27 (29.7)	59 (36.4)	0.277 **
Sanctions for vaccine non-compliance						
Only if established by the Federal Government	37 (24.7)	26 (25.0)		21 (22.8)	42 (25.9)	
States, the Federal District, and municipalities may also establish them	113 (75.3)	78 (75.0)	0.952 **	71 (77.2)	120 (74.1)	0.582 **
Anti-vaccination campaigns						
Must be forbidden	85 (56.7)	57 (55.3)		49 (53.8)	93 (57.4)	
I support them, but with no endorsement of public authorities	49 (32.7)	33 (32.1)		34 (37.4)	48 (29.6)	
I support them, due to the right to freedom of speech	16 (10.6)	13 (12.6)	0.891 **	8 (8.8)	21 (13.0)	0.351 **
Trust in vaccination against COVID-19						
Yes	140 (93.4)	97 (95.1)		87 (94.6)	150 (92.6)	
No, there is no scientific evidence of its effectiveness	5 (3.3)	1 (1.0)		1 (1.1)	5 (3.1)	
No, for different reasons	5 (3.3)	6 (5.9)	0.379 *	4 (4.3)	7 (4.3)	0.720 *
Disclosure of the identity of vaccinated people						
Yes	82 (54.7)	57 (54.8)		50 (54.4)	89 (54.9)	
No	68 (45.3)	47 (45.2)	0.982 **	42 (45.6)	73 (45.1)	0.928 **
Designation of doses for specific groups						
Yes	21 (14.0)	14 (13.5)		12 (13.0)	23 (14.2)	
No	129 (86.0)	90 (86.5)	0.903 **	80 (87.0)	13 (85.8)	0.852 **
Sanctions for those who refuse the vaccine						
Must be pecuniary	131 (88.5)	84 (82.4)		78 (86.7)	137 (85.6)	
Denial of next dose of vaccine	5 (3.4)	5 (4.9)		5 (5.6)	5 (3.1)	
No sanction applied (it is an administrative violation)	12 (8.1)	13 (12.7)	0.366 *	7 (7.7)	18 (11.3)	0.477 *
Person to be punished for vaccine non-compliance						
The citizen	18 (12.0)	13 (12.7)		11 (12.1)	20 (12.4)	
The authority or agency who gave authorization	6 (4.0)	6 (5.9)		2 (2.2)	10 (6.2)	
Both	126 (84.0)	83 (81.4)	0.746 *	78 (85.7)	131 (81.4)	0.380 *
Obstacles to the vaccination against COVID-19						
Public authorities’ speech questioning the effectiveness of the vaccine	69 (47.3)	44 (42.7)		42 (46.1)	71 (45.0)	
People’s distrust in laboratories	13 (8.9)	8 (7.8)		8 (8.8)	13 (8.2)	
Anti-vaccination campaigns	60 (41.1)	44 (42.7)		40 (44.0)	64 (40.5)	
Distrust about the source of the disease	4 (2.7)	7 (6.8)	0.466 *	1 (1.1)	10 (6.3)	0.284 *
Purchase of vaccines by private parties						
I am against it	82 (55.0)	64 (62.7)		57 (64.0)	89 (55.0)	
I am in favor of it	67 (45.0)	38 (37.3)	0.224 **	32 (36.0)	73 (45.0)	0.162 **
Anvisa’s duties to analyze vaccines:						
The agency’s legal and administrative procedures should be followed	109 (73.2)	79 (78.2)		72 (80.9)	116 (72.0)	
Approval by expiration of deadline can be justifiable in case of delays	30 (20.1)	15 (14.9)		13 (14.6)	32 (19.9)	
There may be political intervention in the agency if there is need to speed up the analysis of vaccine proposals	10 (6.7)	7 (6.9)	0.565 **	4 (4.5)	13 (8.1)	0.278 **
Actions of the Judiciary in the context of the COVID-19 vaccination						
The Judiciary branch can interfere in exceptional cases	88 (58.7)	61 (58.7)		54 (58.7)	95 (58.7)	
The Judiciary branch must restrain itself and prioritize administrative decisions	57 (38.0)	38 (36.5)		36 (39.1)	59 (36.4)	
The Judiciary branch can always interfere	5 (3.3)	5 (4.8)	0.851 *	2 (2.2)	8 (4.9)	0.602 *
Use of “immunity passport” in Brazil						
I agree, as long as implemented by federal law	51 (34.0)	43 (42.8)		38 (41.8)	56 (34.6)	
I agree, and any level of the government can establish their own requirements	80 (53.3)	49 (46.7)		46 (50.5)	83 (51.2)	
I disagree	19 (12.7)	11 (10.5)	0.452 **	7 (7.7)	23 (14.2)	0.235 *

Anvisa: Brazilian Health Regulatory Agency.

* Fisher’s exact test;

** Pearson’s chi-squared test.

**Table 4:** Comparison of variables of study (n = 254).

Characteristics	Mandatory vaccination		
	Yes	No	p-value *
	n (%)	n (%)	
Childhood vaccination			
Must be mandatory, even without parental consent	167 (100.0)	0 (0.0)	
Parents’ and guardians’ decision must be respected	54 (63.5)	31 (36.5)	< 0.001
Purchase of vaccines by private parties			
I am against it	138 (94.5)	8 (5.5)	
I am in favor of it	81 (77.9)	23 (22.1)	< 0.001
Trust in vaccination against COVID-19			
Yes	218 (92.8)	17 (7.2)	
No	3 (17.6)	14 (82.4)	< 0.001
Use of “immunity passport” in Brazil			
I agree, as long as implemented by federal law	85 (91.4)	8 (8.6)	
I agree, and any level of the government can establish their own requirements	128 (99.2)	1 (0.8)	
I disagree	8 (26.7)	22 (73.3)	< 0.001

* Person’s chi-squared test.

## Argumentation

This study was the first to analyze the stance of Brazilian federal judges on the implications of the COVID-19 vaccine in the public and private sectors. Our findings show that Brazilian federal judges are aware of the sanitation issues of the country, and that most of them have confident opinions on controversial matters of society, especially the mandatory vaccination of children and adults. The society’s response to crisis - political, sanitary, institutional, among others - is the subject of considerable interest, whether for its voluntary commitment or its engagement in standing up for its convictions.

Vaccine mandates have been a controversial subject throughout the years worldwide. In 1904, the smallpox vaccination campaign in Brazil led to a significant popular riot known as the Vaccine Revolt. This took place in Rio de Janeiro, which was then the capital of Brazil ^13^ ^,^ ^14^. Although the Rio de Janeiro City Ordinances (*Código de Posturas do Município do Rio de Janeiro*) made vaccines mandatory in 1832 ^15^, the regulatory framework for vaccine mandates in Brazil was *Law n. 6,259/1975* ^16^. The Brazilian National Immunization Program (PNI, acronym in Portuguese) was published the following year and established that every citizen should receive mandatory immunization ^17^. In 2004, vaccine schedules were created across the country ^18^. In 2020, at the trial of the *Direct Actions of Unconstitutionality* (ADI, acronym in Portuguese) *6586/DF* ^8^ and *6587/DF* ^19^, the STF ruled that mandatory vaccination does not mean forced vaccination, but that coercive measures can be imposed in any way. Such measures include prohibiting certain activities and denying access to certain spaces, among others, on the condition that these measures are provided for by law ^8^. Table 2 shows that the majority of judges (87.7%) agree with the STF’s decision regarding vaccine mandates. The respondents also consider that the sanctions imposed at those who refuse the vaccine are valid and can be applied by any level of government. The rate of judges who agree with compulsory vaccination is higher than the rate of the general population with the same opinion (79%) ^20^. Among the respondents in favor of vaccine mandates, most of them are also in favor of mandatory vaccination for children and adolescents.

All judges in favor of mandatory vaccination for children and teenagers, with or without parental consent, also support vaccine mandates for the entire population. And most of the judges who believe that vaccination of children and adolescents should require parental consent also agree with vaccine mandates for all citizens (n = 54; 63.5%). The authority of parents over their underage children should not give them permission to jeopardize the health and the life of the latter, regardless of religious or philosophical reasons. Individual autonomy should prevail when legally competent individuals make choices that will not hold a negative impact on the lives of others. This is the case of adult persons who refuse blood transfusions for religious reasons, for instance. However, it is not a prerogative of parents to invoke such rights on behalf of their children, as children are not their property. Therefore, laic and medical values must be considered when dealing with underage children ^8^.

The vaccination of children and teenagers has had mandatory status since 1975 ^16^. Additionally, the Child and Adolescent Statute (*Law n. 8,069/1990*) ^21^ states that it is a duty of the families to secure children and adolescent’ rights to health, including the right to vaccination. Regarding vaccination, choice is not a prerogative, even for adults. Vaccination does not involve one particular person; rather, it impacts not only the health of those around, but also the health of an entire community. Democracy works as a two-way road when it comes to rights and duties. People’s freedom is certainly guaranteed in a democracy, but their duties of responsibility and solidarity must also be considered. “*It is not possible to conceive life in a democracy without responsibility and solidarity with one another*” ^8^.

Behavioral restriction measures are taken to ensure the common good, so that all persons are able to fully live in society. To preserve the health of the community, it is crucial that no person prevents another from their well-being or leads them to an illness. This scenario includes laws related to vaccination, notification, treatment, and isolation of certain diseases, and to the destruction of spoiled food ^22^.

On the other hand, giving the choice to not take a vaccine based on religion or philosophical convictions might lead to an unequal situation in which those who do not have that privilege would be unfairly affected. Considering that all types of vaccines could potentially pose some risk, it would make sense for all persons to be exposed to it. Otherwise, some people would be benefited from population immunity without having taken any risks ^23^.

Trust in vaccination is directly related to favorable opinions and vaccine mandate. Nearly 93% of judges who declared trust in vaccines are also in favor of mandatory immunization (Table 4). The judges’ trust rate is higher than that of the general Brazilian population. A study conducted in January, 2021 - before the authorization for emergency use of the COVID-19 vaccination by Anvisa - showed that over 89% of the respondents were willing to receive a vaccine against the novel coronavirus ^24^.

When comparing the variables of vaccine mandate and federal judges’ level of education, it is possible to notice that education makes no difference in the results concerning their stance in favor of mandatory vaccination against COVID-19. Judges with no specialization or with some specialization degree show an acceptance rate of 88.7%, just slightly higher than that of judges with a postgraduate degree (86.3%) (Table 3). The percentage difference is more significant among the group of judges who agree with vaccine mandates when their experience in the job is considered. Judges with up to 10 years of career (n = 81; 90%) surpass judges with more than 10 years of service (n = 140; 86.3%) by about 4%. Over 59% of judges surveyed in 2021 and 2022 had no specialization or had concluded some specialization degree. The academic profile of Brazilian federal judges is no different from that of other professionals. A study conducted with female judges showed that 59.1% of them had only a Bachelor’s degree or a specialization ^25^. Such results indicate that the level of education is not directly connected to job experience. A possible explanation for that situation is that some people might become judges before starting postgraduate programs.

When it comes to vaccine hesitancy in the context of the COVID-19 vaccination in Brazil, there was little commitment from some of the country’s political authorities. Notoriously fake information from dubious sources was widely disseminated, even by public authorities ^26^. The respondents took those facts into consideration and pointed out that the spread of fake news was an obstacle to the vaccination against COVID-19, as shown in Table 2. Disinformation weakens trust and raises insecurity among people. During the COVID-19 pandemic, all sorts of deliberately wrong information about the risks of vaccinating against the novel coronavirus were repeatedly published in all kinds of media, especially on social media.

The whole concept of vaccination stems from past political tensions (more specifically, the 19th century). Strategies for social equality were employed, whereas there were economic interests from the industry surrounding disease consequences. It is also noteworthy the common belief at the time that some diseases could be “beneficial” to citizens, since they would develop immunity against them ^27^.

Before the implementation of mass immunization programs, people would be vaccinated in the occurrence of imminent epidemic situations or to prevent a significant increase in deaths. They would be vaccinated even without full consent ^27^. The emergence of a new virus and the consequent creation of a new vaccine with unknown outcomes and reactions were expected to raise some resistance to compulsory vaccination. Therefore, campaigns aimed at convincing citizens to get vaccinated should be seen as a necessary means for effective immunization programs.

It is important for society as a whole to be involved in the aforementioned process, including local and regional governments and political and religious leaders. A good example of the involvement of all these parties is the successful smallpox vaccination campaign ^28^.

The spread of false information is powerful and can cause uncontrollable damage. It is worth mentioning the case of a scientific study published by *The Lancet*, which made an implicit connection between the measles, mumps, and rubella vaccine (MMR) and autism, leading thousands of people to oppose that vaccine. The journal did not retract the article until 2010 ^29^.

Vaccination campaigns have also been used for different purposes. There have been campaigns aimed at immunizing bodies that were perceived as a threat to the upper classes or to some economic interests. This is one of the arguments used by those who protest against vaccination ^27^. Vaccine hesitancy is also supported by deliberate negligence towards threats of epidemic or pandemic situations. Before the COVID-19 pandemic outbreak, health agencies in Brazil and worldwide warned people about the risks and recommended preventive measures. On the other hand, vaccine hesitancy goes beyond biomedical debate. A great deal of people take into account the potentially catastrophic social, economic, religious, and moral outcomes of the vaccine, which, according to them, could be worse than the disease itself ^27^. Vaccine hesitancy could also be a consequence of the conflict between individual autonomy and state power. Individual autonomy implies the right of choice, which opposes government power. The point of debate is the prerogatives of the state over the personal lives of citizens ^27^.

Although some arguments suggest that the anti-vaccination propaganda could be backed up by the right to freedom of speech, the respondents do not share the same opinion. Over 56% of them believe in curbing campaigns with such purpose. Despite the majority of judges being against anti-vaccination movements, a significant amount of them is favorable to such movements, showing that there is no consensus on the matter in Brazil.

There is an apparent conflict in the findings regarding trust in vaccination and anti-vaccination movements. Over 93% of federal judges trust vaccines, whereas about 34% of them are favorable to anti-vaccination movements. One possible explanation is that judges take freedom of thought into account, as well as political and/or religious influences. Anti-vaccination activists can be found in any sector of society, regardless of their level of education or their institutional, business, or political stance ^30^.

Besides fake news, about 45% of federal judges suggested that the speech of public authorities questioning the effectiveness of the vaccine was another obstacle to COVID-19 vaccination. According to a previous study, disinformation endorsed by public authorities leads people to vaccine hesitancy. The same study shows that Brazilian people rejected the Chinese vaccine believing that China had deliberately released the virus with the purpose of selling vaccines afterwards ^31^. Community work involving the community, the people, and the government can instill trust in vaccination, thereby reducing vaccine hesitancy. Some authors suggest that media campaigns can raise awareness and encourage vaccination ^32^ despite people’s distrust in information disclosed on the news and on TV, according to a previous study ^33^.

The role of the Judiciary branch has been a constant subject of debate in Brazil when it comes to the COVID-19 pandemic. Some arguments based on the separation of powers affirm that the management of health policies is a duty for the Executive power - with the Brazilian Ministry of Health, the State Health Departments, and the Municipal Health Departments - and therefore should not be a responsibility of the Judiciary branch. Our research with federal judges shows that over 62% of them support the actions of the judiciary branch regarding COVID-19 vaccination. Their opinion is consistent with the modern structure of the separation of powers principle, which defends that branches work independently but also harmoniously. Harmony can be seen in the courtesy among the powers and in the respect for each other’s duties and responsibilities. Full independence of the branches is impractical and unacceptable ^34^, since the checks and balances system work to prevent potential abuses of powers ^35^.

The separation of powers is a system that aims to limit the powers of a leader to avoid concentration of duties in the hands of one single person. In recent years, a variety of matters that could have been more successfully resolved in the political sphere have been brought for consideration by the Judiciary branch. Some of them included rulings regarding the number of city councilors in cities ^36^, the implementation of veto verification in the Brazilian National Congress ^37^, and the legal aspects of same sex marriage ^38^ by the STF.

The typical separation of powers into three branches has undergone adjustments over the years, mostly due to the idea of absolute separation being inconceivable. Peter Hãberle ^39^ affirms that the principle proposed by Montesquieu is open, just as the evolution of the constitutional state is fluid. Concerning social legitimacy, the Legislative and Executive branches tend to have more legitimacy since their representatives are chosen by the people. The Legislative branch represents the diversity of the people and, to some extent, mirrors the people of a nation. It is the appropriate context for political controversy, where the ideas and the causes defended by citizens should be debated. The Executive is responsible for administrative issues and for turning the claims of the people into assertive responses that are supposed to meet their demands. As for the Judiciary branch, it has moved away from its role of merely being la bouche de la loi and now plays a more proactive role ^39^.

The constitutional avoidance doctrine states that the Judiciary branch should only act when strictly necessary. In the case Rescue Army vs. Municipal Court of Los Angeles ^40^, by the U.S. Supreme Court, Justice Wiley Rutledge presented some of the reasons behind the doctrine, such as: (a) the extreme abstractness of constitutional issues; (b) the limitations inherent to the judicial process; (c) the need to respect the decision-making sphere of each branch; and (d) the importance of having a constitutional trial.

One of the most significant adjustments being made to the system of separation of powers is the inclusion of public policy matters into the social rights sphere, most notably public health matters. It is a phenomenon that has been called “the judicialization of politics” or “the politicization of the courts”. That change in paradigm may be the result of a more aggressive or exacerbated activity by the Judiciary branch or the inaction of the other branches. Judicialization is connected to the idea of the effectiveness of constitutional rules ^41^.

In recent decades, there has been a significant increase in requests to the Judiciary branch concerning public health issues (mostly regarding medication not provided for free by the Brazilian Unified National Health System - SUS, acronym in Portuguese). It is understandable that the Judiciary branch take action on public policy matters when the government agencies originally responsible for them refuse to act according to their role, jeopardizing the effectiveness of individual and collective rights ^42^.

The interference of the Judiciary branch in matters related to the COVID-19 pandemic is being questioned. In light of the constitutional avoidance doctrine, Brazilian Justice Nunes Marques has stated that there were no conditions that could justify the action of the Brazilian Judiciary branch in questions regarding vaccination, under the following arguments: (a) there was no COVID-19 vaccine yet when the issue was ruled; (b) the discussion about the efficacy and risks of the vaccine was not being conducted considering purely legal aspects; (c) both the Legislative and Executive branches had suggested solutions to the sanitation issues arising from the COVID-19 pandemic, and this was a political matter, not a legal one; (d) being ruled by the Brazilian court would increase political responsibilities on the matter, which, in turn, would relieve the burden on the agents elected by the people ^8^. Nevertheless, the majority of the STF justices understood that they had the right to act on the matter, as all justifiable conditions were present, in accordance to the direct actions for the *ADI 6586/DF* ^8^ and *ADI 6587/DF* ^19^.

Regarding Anvisa’s role in evaluating and approving the use of vaccines, over 75% of respondents are in favor of giving the agency the prerogative to analyze vaccine proposals. Administrative procedures should not be ignored. Politicizing the matter, or merely considering automatic approval upon expiration of the deadline, could be dangerous to society. According to *Law n. 14,006/2020* ^43^, Anvisa has 72 hours to authorize the import and distribution of health supplies. After that time, the process is automatically authorized. The law also allows the use of the vaccine in the country without prior analysis by Anvisa, as long as it has already been registered by a health agency in the United States, the European Union, Japan, or China. But the specific regulations of each country must also be considered. A vaccine approved for use in China does not necessarily mean it is suitable for use in Brazil. This could undermine Anvisa’s autonomy to make decisions ^44^.

The powers of the republic must respect the scope of agencies of technical expertise. In this sense, the Brazilian National Council of Justice has issued a set of recommendations for observing technical parameters when ruling cases that involve healthcare. One of these recommendations states that the Judiciary branch should not authorize medications to be granted before being registered by Anvisa ^45^ ^,^ ^46^.

The majority of respondents approve the use of immunity passports in Brazil, and this measure is closely related to vaccine mandates. Around 90% of the federal judges who approve the use of immunity passports also support mandatory vaccination against COVID-19 (Table 4). The rate of federal judges supporting the use of immunity passports in Brazil is higher than that of the general population. A previous study shows that 84% of the Brazilian population agrees with the use of immunity passports in the country ^47^. Many countries are currently developing some type of immunity passport that allows people to travel, study, or work without risking other people’s lives. Examples include the “Green Pass program” in Israel, the “Digital Green Certificate” in the European Union, and the Africa Centres for Disease Control and Prevention’s “My COVID Pass” ^48^.

Our study faced some issues regarding the judges’ availability to take part in the survey. The low rate of responses might be due to their fear of taking sides, especially given the state of political polarization that has been taking over the country as well as the anti-science movements and the rise of obscurantism. The place of work of the judges is an aspect that should be analyzed cautiously, as many of them, especially the new ones, usually do not work in their place of birth. For example, a judge from Mâncio Lima, in the state of Acre, could be working in Foz do Iguaçu, in the South of the country.

## Conclusion

Brazilian federal judges who participated in the survey have shown to be aware and up to date with the current discussions involving the COVID-19 vaccine. Most federal judges agree with vaccine mandates for children and adults, believe that any level of government should have the prerogative to impose sanctions on those who refuse to get vaccinated, and disapprove of anti-vaccination movements. There is a direct connection among trust in vaccination, mandatory vaccination, and the requirement of immunity passports, with significant approval rates among respondents. Furthermore, most of them are also in favor of respecting the role of Anvisa and support the intervention of the Judiciary branch in matters of public health policies, including the COVID-19 pandemic.

On the other hand, this study also showed different opinions on controversial matters among respondents. The rate of federal judges who support anti-vaccination movements and who consider the choice of parents over their children’s health demonstrates the legal and ethical complexity of this debate. The potential influence that judges’ convictions might have over their decisions holds an impact on health policies. In this heterogeneous scenario, it is mandatory for the judicial system to find balance in respecting individual liberties without compromising collective responsibilities towards public health. To achieve this, there must be effective communication among the legal community, healthcare agents, and government authorities, so that they can develop clear and coherent norms, firmly based on scientific evidence, with the ability to combat disinformation and ensure that court decisions are made under adequate criteria to deal with health policies. Finally, further research, either with judges from other areas or covering other sanitary crises, is necessary to evaluate their thoughts and perceptions.
